# Supplementary material for: Transcriptomic Insights into Mechanisms of Early Seed Maturation in the Garden Pea (Pisum sativum L.)
Source: Cells. 2020 Mar 23;9(3):779. doi: 10.3390/cells9030779 (PMC7140803; doi:10.3390/cells9030779)
Supplement: Supplementary file 1 [file cells-09-00779-s001.zip › Supplementary materials/FileS4.zip!/FileS3/TFs - Results/Summary Sheets - Modules/K-Means Cluster/Profiles.pdf]

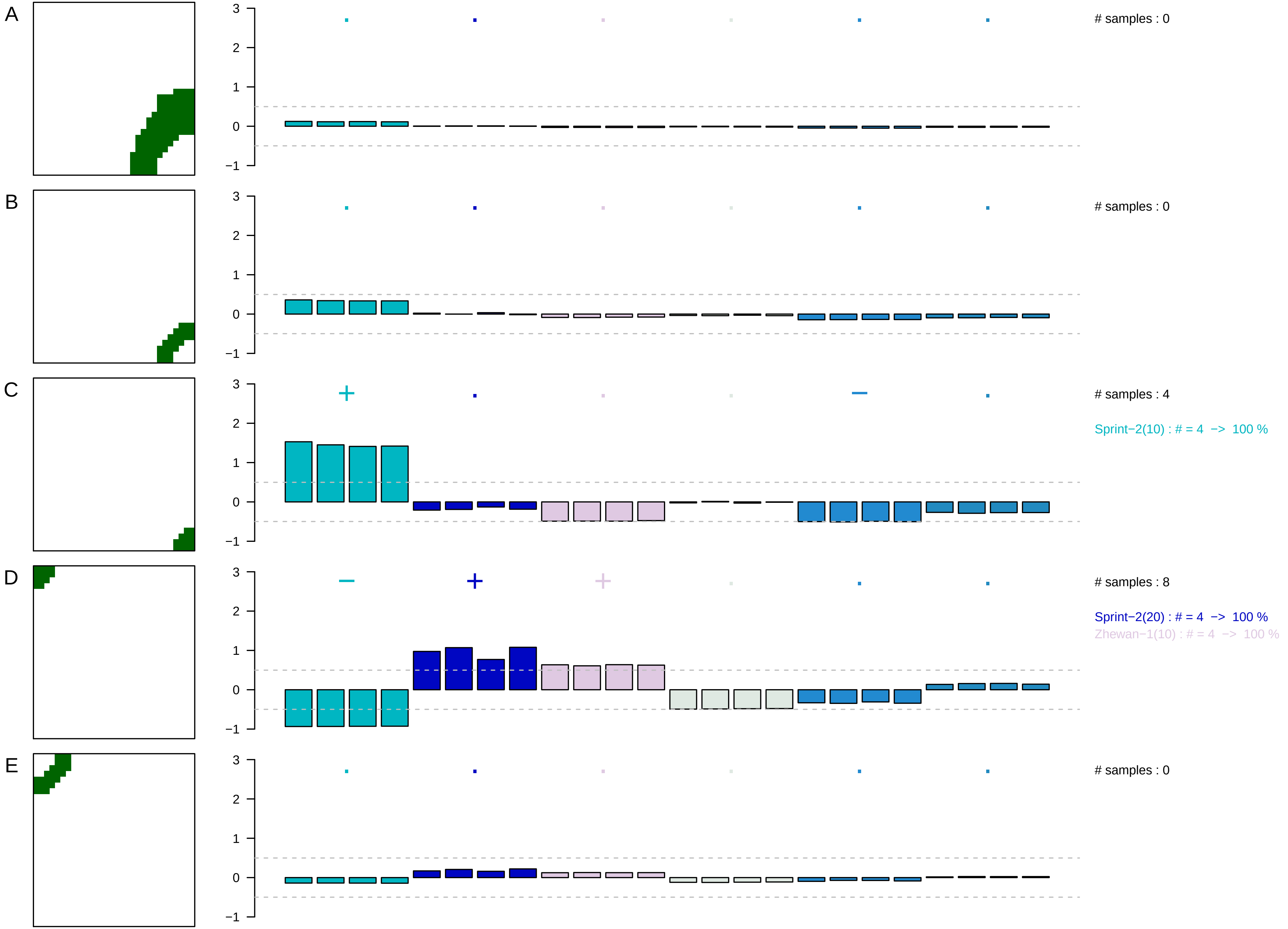
A
B
C
D
E
samples : 0
samples : 0
samples : 4
Sprint−2(10) : # = 4 −> 100 %
samples : 8
Sprint−2(20) : # = 4 −> 100 %
Zhewan−1(10) : # = 4 −> 100 %
samples : 0

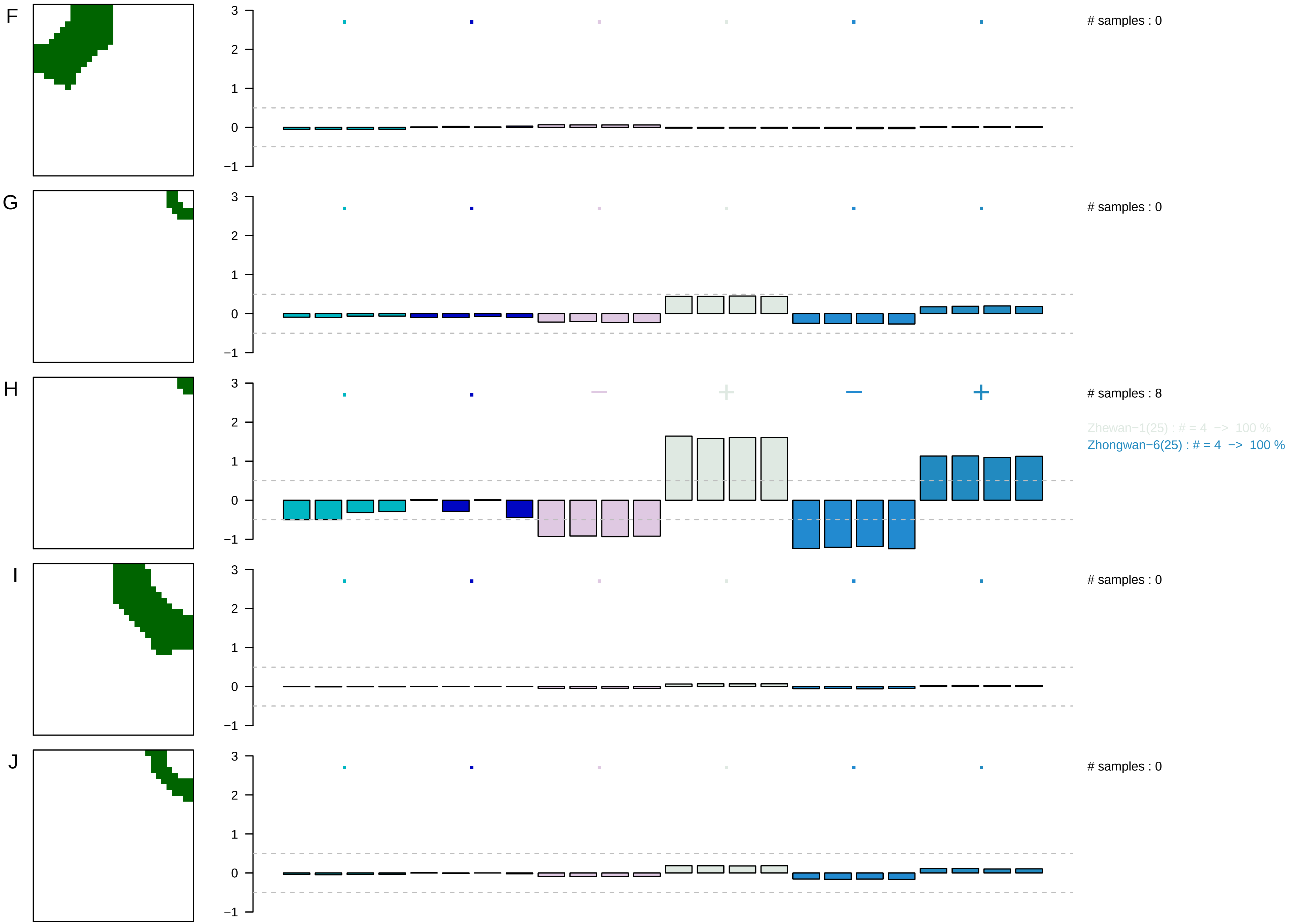
F
G
H
I
J
samples : 0
samples : 0
samples : 8
Zhewan−1(25) : # = 4 −> 100 %
Zhongwan−6(25) : # = 4 −> 100 %
samples : 0
samples : 0

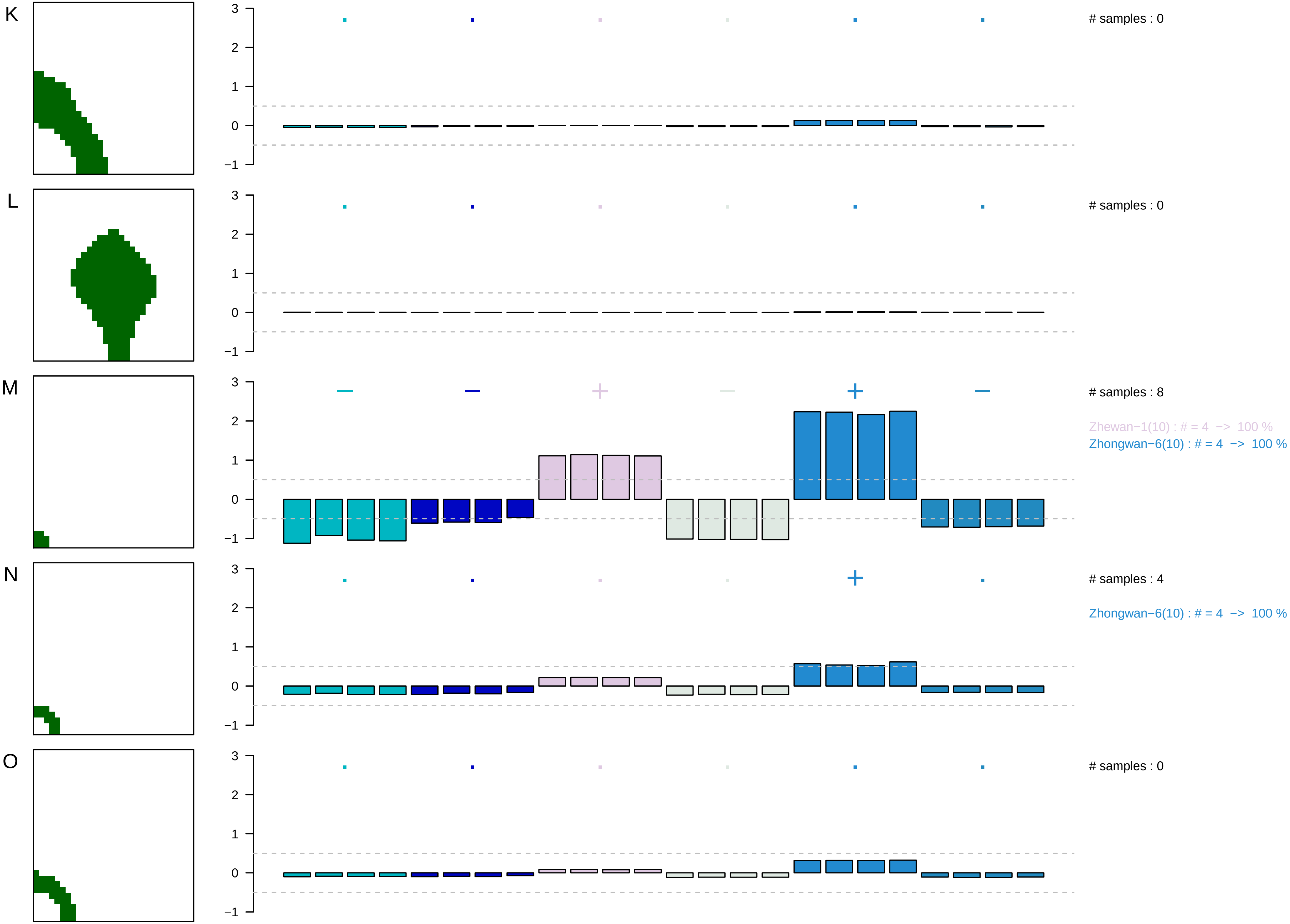
K
samples : 0
L
samples : 0
M
samples : 8
Zhewan−1(10) : # = 4 −> 100 %
Zhongwan−6(10) : # = 4 −> 100 %
N
samples : 4
Zhongwan−6(10) : # = 4 −> 100 %
O
samples : 0
3
2
1
0
−1

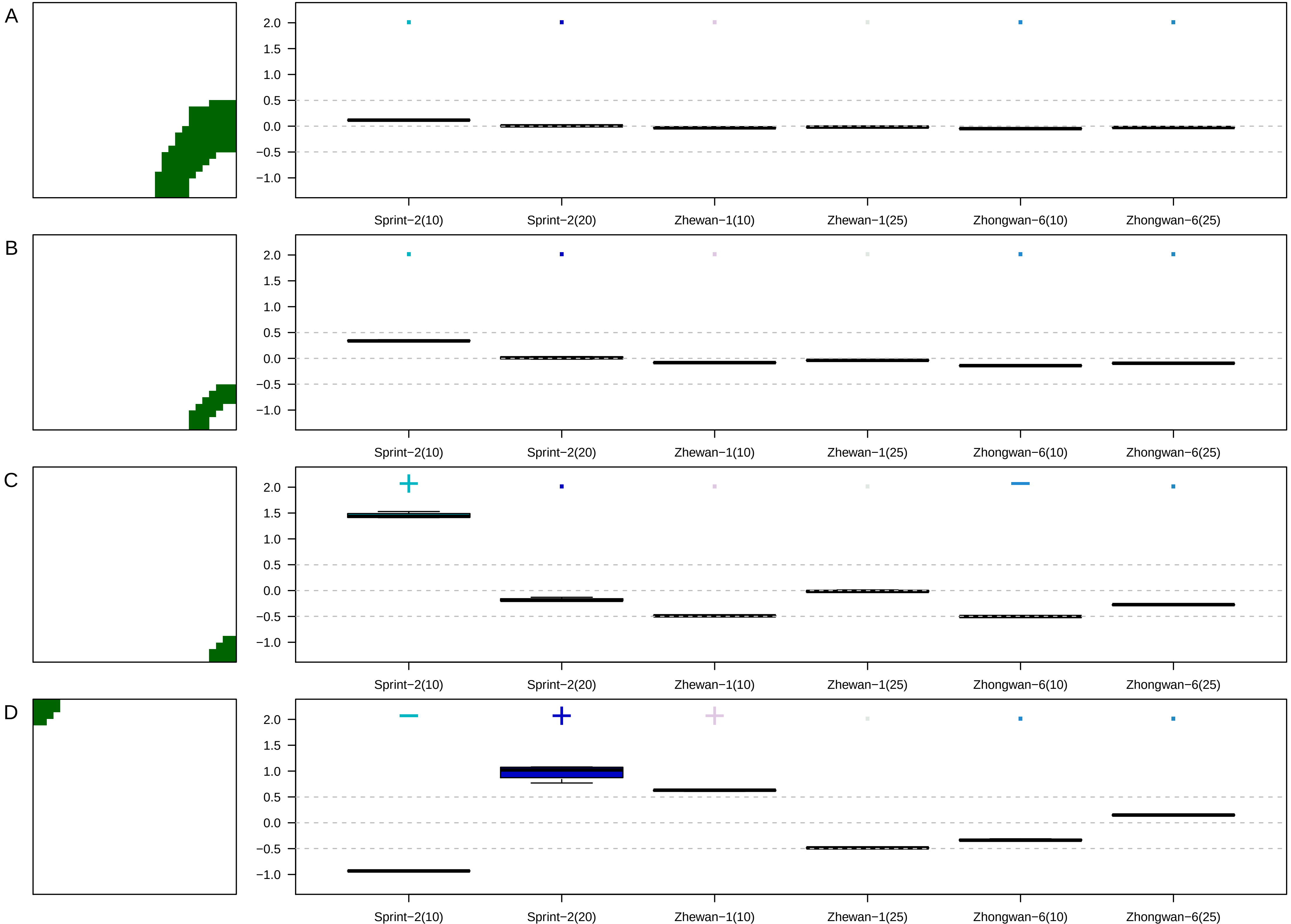
A
B
C
D
2.0
1.5
1.0
0.5
0.0
−0.5
−1.0
Sprint−2(10)
Sprint−2(20)
Zhewan−1(10)
Zhewan−1(25)
Zhongwan−6(10)
Zhongwan−6(25)

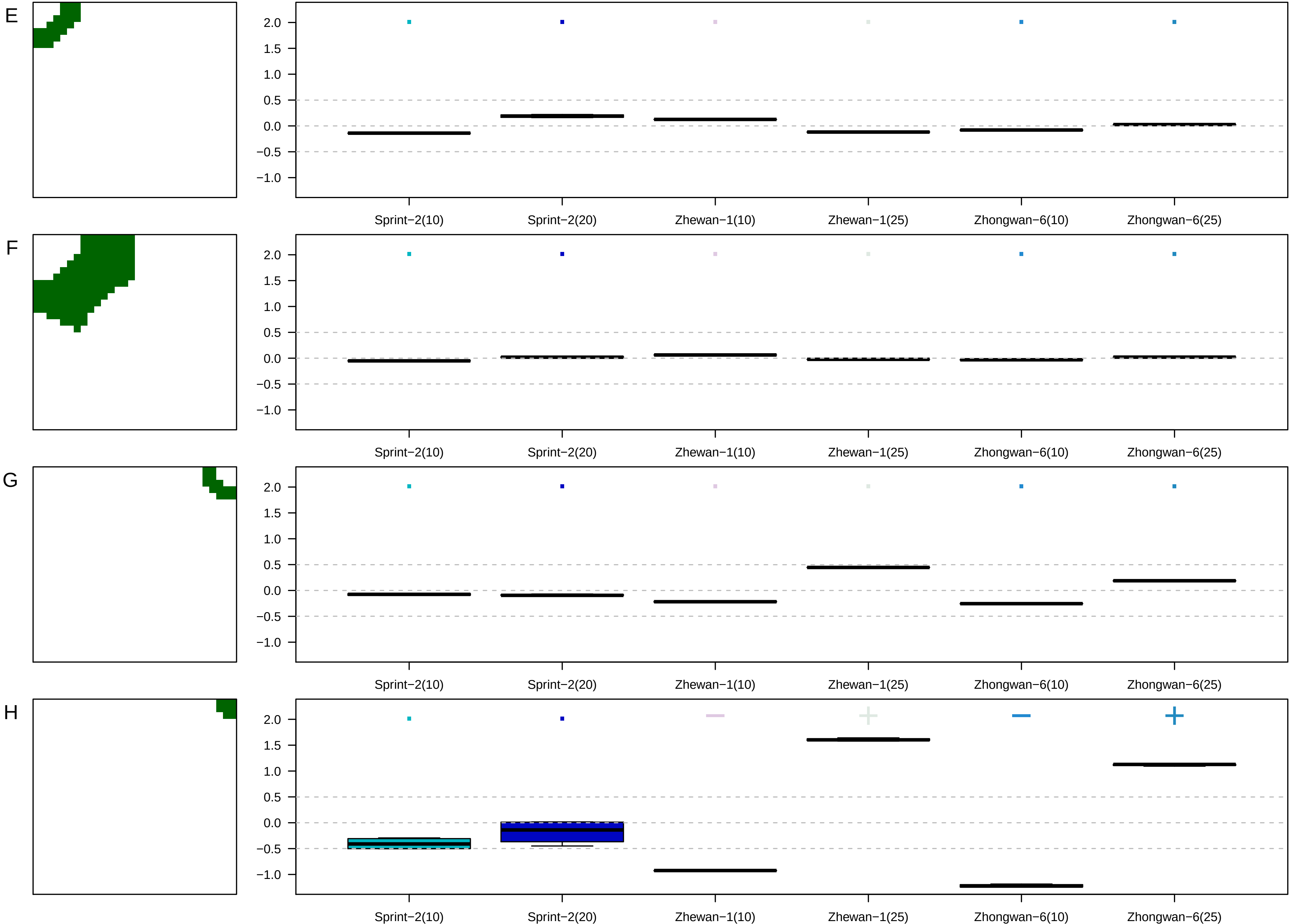
E
F
G
H
2.0
1.5
1.0
0.5
0.0
−0.5
−1.0
Sprint−2(10)
Sprint−2(20)
Zhewan−1(10)
Zhewan−1(25)
Zhongwan−6(10)
Zhongwan−6(25)

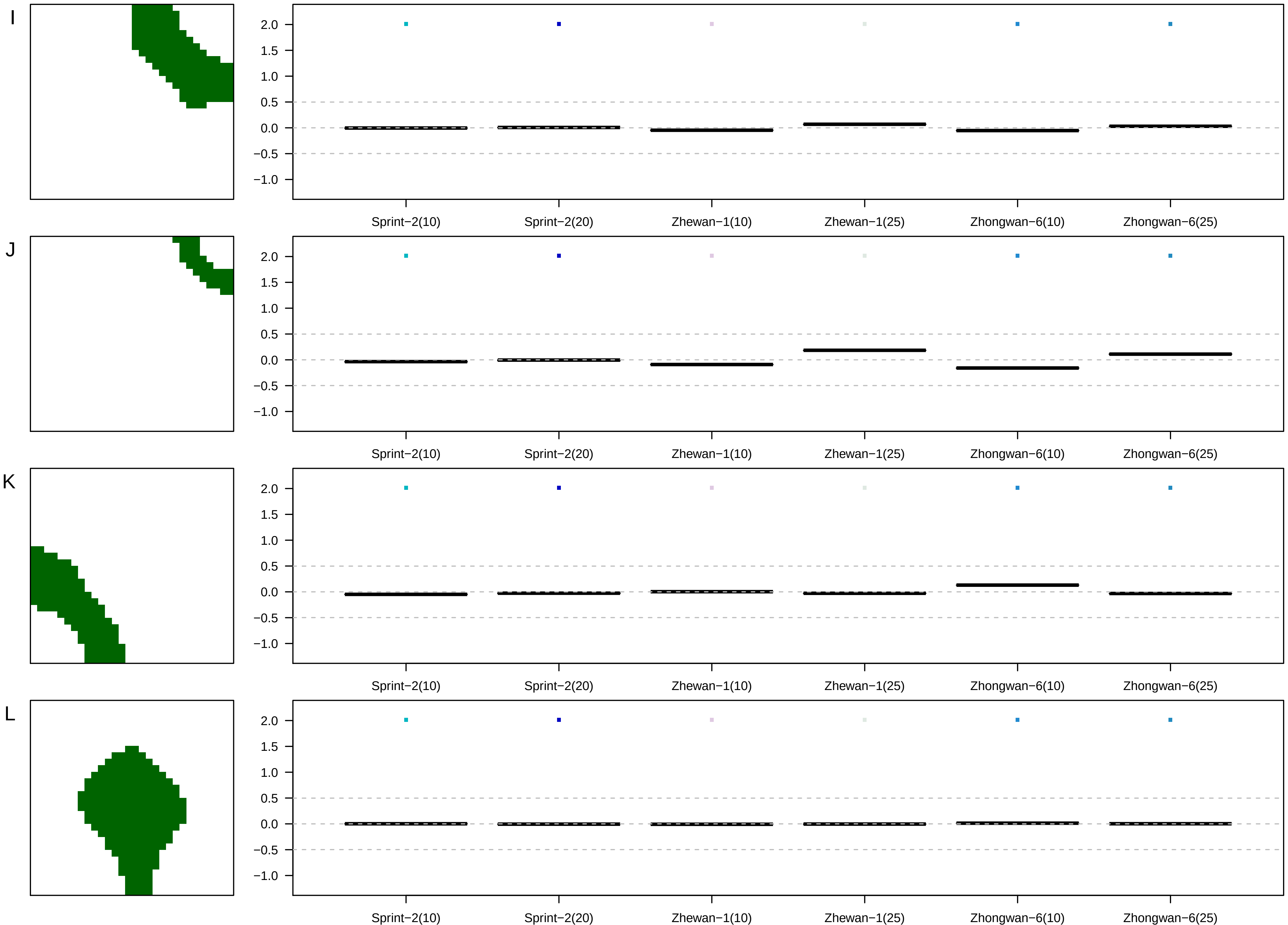
I
2.0
1.5
1.0
0.5
0.0
−0.5
−1.0
Sprint−2(10)
Sprint−2(20)
Zhewan−1(10)
Zhewan−1(25)
Zhongwan−6(10)
Zhongwan−6(25)
J
2.0
1.5
1.0
0.5
0.0
−0.5
−1.0
Sprint−2(10)
Sprint−2(20)
Zhewan−1(10)
Zhewan−1(25)
Zhongwan−6(10)
Zhongwan−6(25)
K
2.0
1.5
1.0
0.5
0.0
−0.5
−1.0
Sprint−2(10)
Sprint−2(20)
Zhewan−1(10)
Zhewan−1(25)
Zhongwan−6(10)
Zhongwan−6(25)
L
2.0
1.5
1.0
0.5
0.0
−0.5
−1.0
Sprint−2(10)
Sprint−2(20)
Zhewan−1(10)
Zhewan−1(25)
Zhongwan−6(10)
Zhongwan−6(25)

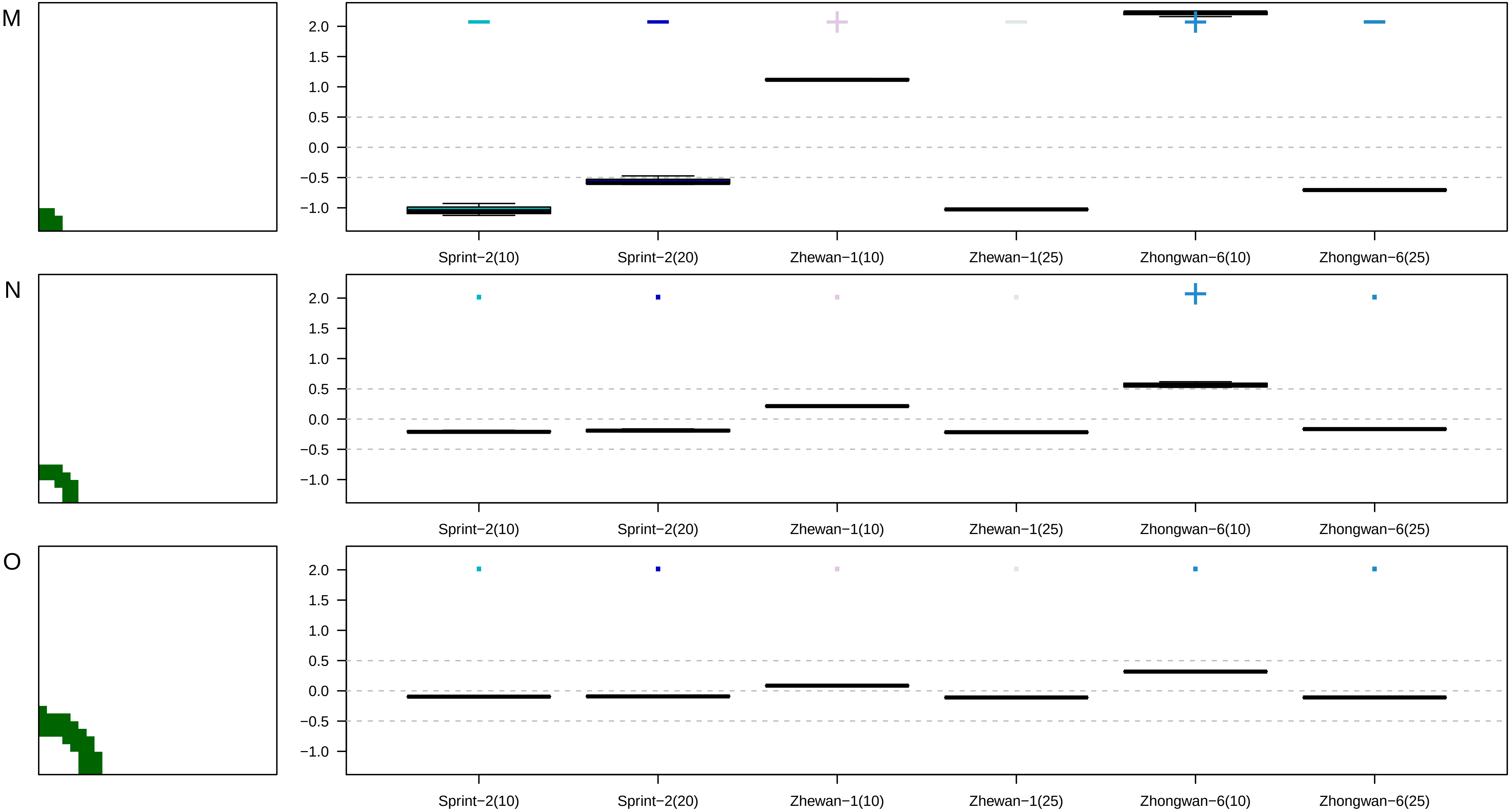
M
N
O
2.0
1.5
1.0
0.5
0.0
−0.5
−1.0
Sprint−2(10)
Sprint−2(20)
Zhewan−1(10)
Zhewan−1(25)
Zhongwan−6(10)
Zhongwan−6(25)
